# RIP1 Perturbation Induces Chondrocyte Necroptosis and Promotes Osteoarthritis Pathogenesis *via* Targeting BMP7

**DOI:** 10.3389/fcell.2021.638382

**Published:** 2021-04-16

**Authors:** Jin Cheng, Xiaoning Duan, Xin Fu, Yanfang Jiang, Peng Yang, Chenxi Cao, Qi Li, Jiying Zhang, Xiaoqing Hu, Xin Zhang, Yingfang Ao

**Affiliations:** Beijing Key Laboratory of Sports Injuries, Department of Sports Medicine, Institute of Sports Medicine of Peking University, Peking University Third Hospital, Beijing, China

**Keywords:** cartilage, chondrocyte, osteoarthritis, necroptosis, receptor-interacting protein kinase 1, extracellular matrix

## Abstract

Osteoarthritis (OA) is a highly prevalent and debilitating joint disorder that characterized by progressive destruction of articular cartilage. There is no effective disease-modifying therapy for the condition due to limited understanding of the molecular mechanisms on cartilage maintenance and destruction. Receptor-interacting protein kinase 1 (RIP1)-mediated necroptosis plays a vital role in various diseases, but the involvement of RIP1 in OA pathogenesis remains largely unknown. Here we show that typical necrotic cell morphology is observed within human OA cartilage samples *in situ*, and that RIP1 is significantly upregulated in cartilage from both OA patients and experimental OA rat models. Intra-articular RIP1 overexpression is sufficient to induce structural and functional defects of cartilage in rats, highlighting the crucial role of RIP1 during OA onset and progression by mediating chondrocyte necroptosis and disrupting extracellular matrix (ECM) metabolism homeostasis. Inhibition of RIP1 activity by its inhibitor necrostatin-1 protects the rats from trauma-induced cartilage degradation as well as limb pain. More importantly, we identify bone morphogenetic protein 7 (BMP7) as a novel downstream target that mediates RIP1-induced chondrocyte necroptosis and OA manifestations, thereby representing a non-canonical regulation mode of necroptosis. Our study supports a model whereby the activation of RIP1-BMP7 functional axis promotes chondrocyte necroptosis and subsequent OA pathogenesis, thus providing a new therapeutic target for OA.

## Introduction

Osteoarthritis (OA) is the most common joint disease, which is a major source of pain, disability, and socioeconomic cost worldwide (Hunter et al., 2014; Safiri et al., 2020). The epidemiology of OA is complex and multifactorial, with genetic, biological, and biomechanical components (Glyn-Jones et al., 2015; Martel-Pelletier et al., 2016). Cartilage destruction is a key feature of OA, but current treatment options are limited to symptoms relief, with no effective disease-modifying OA drugs (DMOADs) discovered so far (Bijlsma et al., 2011). Therefore, unraveling novel molecular mechanisms of cartilage maintenance and destruction is likely to yield new therapeutic strategies for OA.

Loss of chondrocyte cellularity within the articular cartilage is one of the prominent events that contribute to its degradation. However, what controls the fate of chondrocytes during OA pathogenesis is still uncertain. Chondrocyte death has been shown to play a vital part in OA. Previous studies mainly focused on chondrocyte apoptosis rather than necrosis, which has long been regarded as an unregulated form of passive cell death and cannot be used as a therapeutic target (Ryu et al., 2012; Hosseinzadeh et al., 2016). Recent advances have identified a “programmed” form of necrosis (i.e., necroptosis), which has been placed in a central position in the pathogenesis of various diseases. Necroptosis is mediated by necrosome, a supermolecular complex which contains receptor-interacting protein kinase 1 and 3 (RIP1, RIP3), and its direct substrate mixed-lineage kinase domain-like protein (MLKL), targeting the complex to appropriate downstream effectors in the necroptosis-inducing process (Cho et al., 2009; He et al., 2009; Wang et al., 2014). Previous studies have discovered a possible link between necroptotic process and cartilage injury depending on oxidative stress and cytokine release in OA, and the TRIM24-RIP3 axis was proposed to promote OA chronicity by modulating the expression of catabolic factors (Riegger and Brenner, 2019; Jeon et al., 2020; Stolberg-Stolberg et al., 2020). However, the involvement of RIP1 during OA pathogenesis still lacks direct evidence. Unlike RIP3 primarily mediating necroptosis, RIP1 induces both necroptosis and apoptosis when appropriate downstream signals are present, which makes it a major regulator in cell death (Dannappel et al., 2014; Degterev et al., 2019). And given the fact that RIP1 functions upstream of RIP3 and MLKL, it is likely that RIP1 might be a more effective therapeutic target for clinical treatment of OA.

The cellular events acting downstream of the necrotic signaling complex to execute necroptosis depends on the cell type and stimulus. Therefore, further investigation is required for complete understanding of tissue-specific intracellular necroptosis mediators during OA. A recent report demonstrated that knockdown of RIP1 protected chondrocytes against inflammation and apoptosis induced by interleukin (IL)-1β in a manner mediated by a TRIF/MyD88-RIP1-TRAF2 negative feedback loop (Liang et al., 2019). Although this observation suggested a possible role of RIP1 in cartilage degeneration, the physiological and pathological functions of RIP1 in chondrocytes and the underlying mechanism have not yet been fully addressed. The contribution of RIP1-mediated necroptosis during OA onset and progression *in vivo* is also undefined. Herein, we aim to investigate the possible functions and underlying molecular mechanisms of RIP1 in OA pathogenesis.

In this study, we provide the first *in situ* evidence that typical morphological features of necroptosis occur in chondrocytes within osteoarthritic human cartilage, and demonstrate that the expression level of RIP1 is significantly upregulated in cartilage from both OA patients and experimental OA rat models. We are also the first to prove that intra-articular RIP1 overexpression is sufficient to induce OA manifestations in rats, highlighting the crucial role of RIP1 at OA onset by mediating chondrocyte necroptosis and extracellular matrix (ECM) degradation. And inhibition of RIP1 activity by its inhibitor necrostatin-1 (Nec-1) protects the rats from trauma-induced cartilage disruption as well as limb pain. More importantly, we demonstrate that MLKL is dispensable for RIP1-induced chondrocyte necroptosis and OA pathogenic signatures, and identify bone morphogenetic protein 7 (BMP7) as a novel downstream target of RIP1 in chondrocytes, thereby representing a non-canonical regulation mode of necroptosis. Our study supports a model whereby RIP1-BMP7 functional axis participates in the regulation of OA pathogenesis, thus providing a new therapeutic target for the clinical treatment of OA.

## Materials and Methods

### Human Cartilage Samples

Human OA cartilage samples were obtained from individuals undergoing total knee arthroplasty in Peking University Third Hospital (Supplementary Table 1). All patients provided written informed consent. Sample collection was approved by the ethics committee of the hospital. Healthy human cartilage samples were obtained from Shanxi Osteorad Biomaterial Co., Ltd., provided by donors. The inclusion and exclusion criteria for screening OA patients are as follows:

•Inclusion criteria (all of the following):

1. Age of above 55 years old;
2. Plain X-rays showing OA (Kellgren and Lawrence gradus 3–4) which correlates with clinical symptoms;
3. Severe bone destruction and significantly narrowed joint space indicated by X-rays, or varus and valgus deformity/flexion contracture deformity, which has severely affected joint mobility and living ability of the patients;
4. Symptoms were not improved by conservative treatment.

•Exclusion criteria (any of the following):

1. No symptom of pain or deformity;
2. Paralysis of the tissues around the knee joint;
3. Severe cardiovascular disease;
4. Acute or chronic infectious diseases;
5. Hemorrhagic disorders;
6. Pulmonary insufficiency;
7. Mental instability.

### Experimental OA Rat Models

To produce experimental OA models, 10 weeks old male Sprague–Dawley (SD) rats were subjected to surgical anterior cruciate ligament transection (ACLT), female rats were not used because of the effects of female hormones on OA pathogenesis. Briefly, under general anesthesia, the anterior cruciate ligament of the right knees was transected. Three days after surgery, the rats were randomly divided into three groups: ACLT + DMSO group, ACLT + Nec-1 L (0.025 mg/kg) group and ACLT + Nec-1 H (0.05 mg/kg) group. And sham operation was performed on control rats. Nec-1 was diluted in 50 μL of DMSO and injected into the articular twice a week, and continued for 4 weeks until the rats were sacrificed. Spontaneous OA in rats was examined at 12 months of age. All animals were maintained in the Animal Facility of Peking University Health Science Center, and ethical approval was obtained from the Institutional Animal Care and Use Committee.

### Primary Culture of Chondrocytes

Primary rat chondrocytes were isolated from femoral condyles and tibial plateaus of SD rats weighing 80 g by digesting cartilage tissue with 0.25% trypsin for 30 min and then 0.2% type II collagenase for 4 h at 37°C. The cells were suspended in Dulbecco’s Modified Eagle’s Medium (DMEM; Gibco) supplemented with 10% fetal bovine serum (FBS; HyClone) and antibiotics (penicillin G and streptomycin). All cells were maintained as a monolayer in a humidified incubator containing 5% CO_2_ at 37°C. Human chondrocytes were isolated from healthy and OA human cartilage samples using the same method. Mouse chondrocytes were isolated from wild-type and *Mlkl* gene knockout (KO) mice which were generated as previously described (He et al., 2009). Recombinant IL-1β, TNF-α and BMP7 used to treat chondrocytes were purchased from PeproTech, Inc.

### Adenovirus Infection of Chondrocytes and Intra-Articular Injection of Rats

Chondrocytes were cultured for 2 days, infected with replication-defective adenovirus encoding the complete *Rip1* open reading frame (Ad-*Rip1*) or adenoviral vector (Ad-Ctl) at the indicated multiplicity of infection (MOI), and cultured in the absence or presence of Nec-1. For intra-articular injection of the adenovirus, SD rats weighing 80 g were randomly divided into four groups: Healthy control group, Ad-Ctl group [5 × 10^8^ plaque forming units (pfu)], Ad-*Rip1* L group (10^8^ pfu) and Ad-*Rip1* H group (5 × 10^8^ pfu). The adenovirus was diluted in 50 μL of physiological saline and injected into the knee joints of rats through the patellar ligament using a 26-gauge needle once per week. The rats were sacrificed 4 weeks after the initial injection under anesthesia. All animals were maintained in the Animal Facility of Peking University Health Science Center, and ethical approval was obtained from the Institutional Animal Care and Use Committee.

### Evans Blue Dye Staining Assay

For human OA cartilage tissues, freshly obtained samples were soaked in Evans blue dye (EBD) dissolving in saline (1 mg/mL) overnight, and Hoechst 33342 (2.5 μg/mL) was added 10 min before harvest. The fluorescence was then visualized by confocal microscopy.

For rats with intra-articular injection of Ad-Ctl or Ad-*Rip1*, EBD (100 mg/kg) was intraperitoneally injected 1 day before sacrifice, and then the cartilage was snap-frozen and embedded in O.C.T. compound. Frozen cryosections (7 μm thickness) were mounted onto slides, fixed in ice-cold methanol for 5 min, and then incubated with Hoechst 33342 for 10 min. After washed with PBS, the slides were visualized under confocal microscope.

### Cell Viability Assay

Chondrocyte necrosis was determined by lactate dehydrogenase (LDH) assay. The LDH concentration in the culture medium was spectrophotometrically assayed using a kit from Sigma (MAK066) according to the manufacturer’s instructions. CCK-8 and Live-Dead staining assay was used to measure cell viability. For CCK-8 assay, 10 μL of CCK-8 (Dojindo) was added into each well of culturing cells, and after 1 h of incubation, the absorbance was measured at 450 nm using the microplate reader. Background reading of medium was used to normalize the result. For Live-Dead staining, the culture medium of chondrocytes was replaced by a solution of PBS containing 1 mg/mL calcein-AM (Thermo Fisher Scientific) and 1 mg/mL propidium iodide (PI) (Thermo Fisher Scientific). And the stained cells were observed under confocal microscope after 30 min of incubation.

### Transmission Electron Microscopy

Human cartilage tissues were cut into 1 mm^3^ pieces and fixed immediately in 2.5% glutaraldehyde at 4°C for 2 h, dehydrated in a graded ethanol series, embedded and sectioned at a thickness of 50 nm. The sections were stained with uranium acetate for 30 min and lead citrate for 5 min, and then examined with transmission electron microscope (TEM) (JEM1400PLUS, JEOL).

### Histological Assessment

Human and rat cartilage tissues from the knee joints were resected and fixed in 4% paraformaldehyde, decalcified in 0.5 M EDTA, dehydrated in a graded ethanol series and embedded in paraffin. The paraffin blocks were sectioned at a thickness of 5 μm. The sections were deparaffinized in xylene, and cartilage destruction in rats was examined using safranin O and fast green (Sigma) staining and scored by two blinded observers using the Osteoarthritis Research Society International (OARSI) histological grading scale (Moskowitz, 2006; Pritzker et al., 2006). Immunohistochemistry (IHC) analysis was performed with antibodies recognizing type II collagen (Abcam, ab34712; 1:200), MMP13 (Abcam, ab219620; 1:200), RIP1 (BD Biosciences, 610459; 1:200) and RIP3 (Novusbio, NBP1-77299; 1:200). HRP conjugated anti-mouse and anti-rabbit secondary antibodies were purchased from ZSGB-BIO.

### Immunofluorescence

Rat chondrocytes were treated with or without 10 ng/mL IL-1β for 24 h, rinsed in PBS and fixed with 4% paraformaldehyde for 15 min at room temperature. Triton X-100 was used to penetrate the cell membrane for 5 min, and goat serum was applied to block non-specific binding sites. Then the cells were incubated with primary antibodies against RIP1 (BD Biosciences, 610459; 1:200) at 4°C overnight, washed with PBS and incubated with anti-mouse IgG (Alexa Fluor^®^ 488) (Abcam, ab150113; 1:1,000). Nuclei were counterstained with Hoechst 33342 for 10 min. After washed again, the samples were visualized using confocal microscope.

### Quantitative Real-Time PCR

Total RNA was extracted using TRIzol reagent (Invitrogen). Isolated RNA was reverse-transcribed using RevertAid First Strand cDNA Synthesis Kit (Fermentas, K1622) according to the manufacturer’s instruction, and quantitative real-time PCR (qRT-PCR) was performed using StepOne Plus Real-Time PCR System (Applied Biosystems). Primers for qRT-PCR were shown in Supplementary Table 2. Amplification was performed as follows: 95°C for 2 min, followed by 40 cycles of 95°C for 15 s and 60°C for 30 s. A dissociation stage was added at the end of the amplification procedure. The expression level of GAPDH was used as an internal control. The relative expression level was calculated by 2^–Δ^ ^*CT*^.

### Annexin V/PI Staining Assay

Live, apoptotic and necrotic chondrocytes were distinguished by Alexa Fluor^®^ 488 annexin V/Dead Cell Apoptosis Kit (Invitrogen) according to the manufacturer’s instruction. Briefly, cells were gently trypsinized, washed with serum-containing media and collected by centrifugation. Then the cell pallets were resuspended in 500 μL of 1x binding buffer. After adding 5 μL of annexin V-FITC and 5 μL of PI, the samples were incubated at room temperature for 5 min in the dark and proceeded for quantification by flow cytometry within 1 h. Unstained cells were used as a negative control.

### Cell Cycle Analysis

Chondrocytes were harvested, washed in PBS and fixed in 75% ethanol at 4°C overnight. Then the cells were washed and centrifuged at 850 *g*, the supernatant was discarded and 50 μL of a 100 μg/mL RNase stock was added. After incubated at 37°C for 30 min, the cells were centrifuged and resuspended in 450 μL of PBS. After adding 50 μL of a 50 μg/mL PI stock solution and incubated for 10 min in the dark, the samples were proceeded for flow cytometric analysis.

### Western Blotting and Enzyme-Linked Immunosorbent Assay

Western blotting analysis was conducted using the protein of lysates isolated from cultured rat chondrocytes. The cell lysates prepared in lysis buffer (150 mM NaCl, 1% NP-40, 50 mM Tris, 0.2% SDS, 5 mM NaF) containing protease inhibitors (Roche) were centrifuged, and the supernatants were separated by SDS-PAGE and blotted on a polyvinylidene fluoride membrane (Bio-Rad). After incubated with specific antibodies, proteins were detected using BIO-RAD ChemiDoc XRS + system. Antibodies direct against the following proteins were used: cleaved poly ADP-ribose polymerase (PARP) (Abcam, ab32064; 1:1,000), cleaved caspase-3 (Abcam, ab32042; 1:500), MMP1 (Proteintech, 10371-2-AP; 1:1,000), MMP13 (Abcam, ab39012; 1:2,000), IL6 (Abcam, ab9324; 1:800), type II collagen (Abcam, ab34712; 1:1,000), SOX9 (Abcam, ab185966; 1:1,000), RIP1 (BD Biosciences, 610459; 1:1,000), RIP3 (Novusbio, NBP1-77299; 1:1,000), and BMP7 (Bioss, bs-2242R; 1:1,000). Anti-GAPDH and HRP-conjugated secondary antibodies were purchased from ZSGB-BIO.

Rat articular chondrocytes were infected with Ad-Ctl or Ad-*Rip1* at the indicated MOI, after 48 h of culture, the amount of secreted BMP7 in the cultural supernatant was determined by the enzyme-linked immunosorbent assay (ELISA) Development kit (SEA799Ra, Cloud-Clone) according to the manufacturer’s suggested protocol.

### Cartilage Explants Experiment

Cartilage disk from intact human knee cartilage was cut into pieces of approximately 1 mm^3^ in volume, and each piece was cultured in medium supplemented with Ad-Ctl (100 MOI), Ad-*Rip1* or Ad-*Rip1* with 50 μM Nec-1 for a total of 10 days. The cartilage piece cultured in medium without supplement was used as negative control. Then the explants were proceeded for the measurement of glycosaminoglycan (GAG) content, or fixed, cut into sections and stained with toluidine blue. The cultural supernatant was collected for the quantification of secreted cartilage oligomeric matrix protein (COMP) by using the ELISA Development kit (abx256440, Abbexa).

### Measurement of GAG Content

The cartilage explants were grinded and digested overnight in papainase (125 μg/mL) at 60°C. The GAG content was measured using a DMMB assay. Briefly, 20 μL of lysates were mixed with 200 μL of DMMB working solution for 30 min at room temperature. The absorbance was then measured at 525 nm. Chondroitin sulfate (Sigma) was used as a standard.

### Alcian Blue and Alizarin Red Staining

Chondrocytes were fixed with 4% paraformaldehyde in PBS for 15 min at room temperature and washed with diH_2_O three times, then 1% Alcian blue staining solution (Cyagen) or 2% Alizarin red staining solution (Sciencell) was added. After 30 min of incubation, cells were washed and images were taken using optical microscope.

### Nanoidentation Assessment

Biomechanical properties of rat cartilage surface were analyzed using nanoindentation. Cartilage samples were isolated from the central part of rat femoral condyle. Circumfluent PBS solution was used to maintain hydration. All indentations were performed using TI 950 TriboIndenter with a 400 μm radius curvature conospherical diamond probe tip. A trapezoidal load function was applied to each indent site with 10 s peak load, 2 s hold, and 10 s unload. Indentations were force-controlled to a maximum indentation depth of 500 nm. The microscopic geomorphology of the indentation zones was captured using micro-scanning apparatus.

### Hotplate Analysis

Hotplate test was applied to analyze the pain response of rat limbs. The rats were placed on the hotplate (UGO BASILE) setting at 55°C. The latency period for hind limb response (e.g., shaking, jumping, or licking) was recorded as response time. Each rat was measured for three times. The observers were blinded to the animal group.

### Weight Bearing Test

The weight distribution of hind paws of rats was measured using the incapacitance tester (UGO BASILE). Rats were standing inside the chamber with each hind paw on one transducer during testing. The duration time was set for 9 s. The results were shown as the ratios of weight placed on the injected/operated (right) hindlimb vs. that on the contralateral sham (left) hindlimb. Measurements were taken for three times for each rat. The observers were blinded to the animal group.

### Terminal Deoxynucleotidyl Transferase dUTP Nick-End Labeling Assay

Transferase dUTP nick-end labeling (TUNEL) assay was performed using a TUNEL Apoptosis Assay Kit (HRP-DAB, Beyotime, C1098) for *in situ* detection of apoptotic cells in rat cartilage tissues according to the manufacturer’s instructions. Briefly, the paraffin slices were deparaffinized, hydrated, incubated with 20 μg/mL Proteinase K at 37°C for 20 min, washed with PBS and then incubated with 3% H_2_O_2_ in PBS at 25°C for 20 min. After washed again, the slices were incubated with a terminal deoxynucleotidyl transferase (TdT) enzyme working solution at 37°C in the dark for 60 min, washed and incubated with Streptavidin-HRP solution at 25°C for another 30 min, and then with a DAB working solution at 25°C for 5 min. After stop the reaction by washing with PBS, the slices were dehydrated, mounted and examined using optical microscope.

### Microarray Analysis

Rat articular chondrocytes were infected with Ad-Ctl or Ad-*Rip1* at a MOI of 100 for 24 h. Total RNA was extracted from each group using Trizol reagent. The cDNA libraries were then constructed and quantified with the Agilent Bioanalyzer 2100 system. Sequencing was performed using the Illumina HiSeq platform (Agilent Technologies, United States) by Novogene Co., Ltd., and 150 bp paired-end reads were generated. Analysis of differentially expressed genes was performed using the DEGseq R package. Gene ontology (GO) analysis was performed to facilitate elucidating the biological implications of the differentially expressed genes, including biological process (BP), cellular component (CC), and molecular function (MF) (Ashburner et al., 2000). Pathway analysis was used to identify the significantly influenced pathways on which the differentially expressed genes have affected according to the Kyoto Encyclopedia of Genes and Genomes (KEGG) database (Kanehisa and Goto, 2000). Fisher’s exact test was applied to identify significant GO categories as well as influenced pathway. And the threshold of significance was defined by *P* value (Draghici et al., 2007). Enrichment maps were generated using Clusterprofiler for graphical representation of top 30 enriched biological processes and KEGG pathways, including upregulated and downregulated ones.

### RNA Interference-Mediated Gene Silencing

For gene-silencing assays, siRNAs 19 nucleotides in length with a dTdT overhang at the 3′ terminus were designed to target BMP7, the siRNA sequences were as follows: siRNA1, 5′-CCATCGAGAGTTCCGGTTT-3′; siRNA2, 5′-GGATCTATAAGGACTACAT-3′; siRNA3, 5′-GGAGGGCTGGTTGGTATTT-3′. Rat chondrocytes were seeded onto 6-well plates and cultured for 24 h, then transfected with 3 μL of siRNA (10 μM) using Lipofectamine RNAiMAX (Invitrogen) following the manufacturer’s instructions and harvested after 48 h.

### Statistical Analysis

Results were presented as the mean ± standard error of the mean (SEM). GraphPad Prism 7 was used for statistical analysis. Statistical significance with parametric data was assessed by Student’s *t*-tests (two groups), one-way ANOVA (homogeneity of variance, three or more groups), or non-parametric test (uneven variance). Significance was accepted at the 0.05 level of probability (*P* < 0.05).

## Results

### RIP1 Is Upregulated in OA Cartilage, in Which Chondrocyte Necrosis Is Observed

**FIGURE 1:**
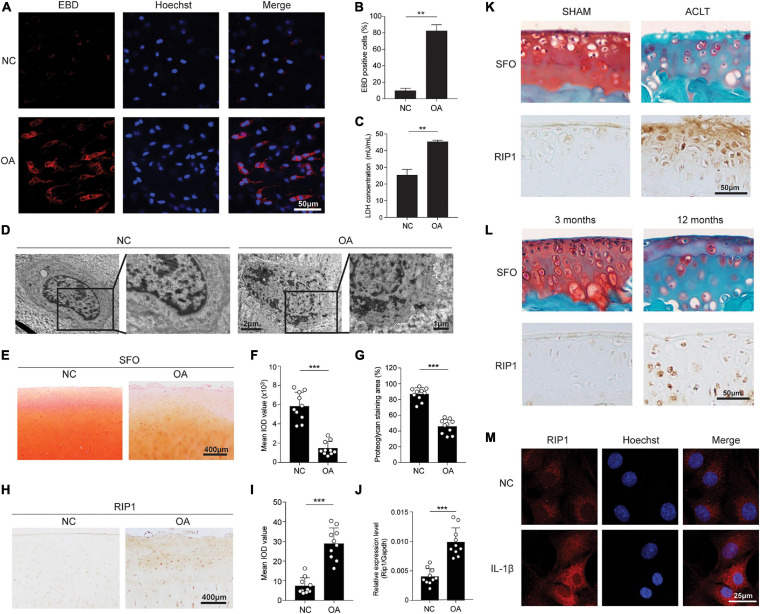
Chondrocyte necrosis and RIP1 upregulation are both presented in OA cartilage tissues. **(A,B)** Representative photomicrographs **(A)** and quantitative data **(B)** of chondral EBD uptake (red) in healthy and OA human cartilage (*n* = 6 for each group). Cell nuclei were labeled by Hoechst. **(C)** LDH concentrations in the cultural supernatant of healthy and OA human cartilage explants cultured *ex vivo* for 10 days (*n* = 5 for each group). **(D)** Representative TEM images of healthy and OA human cartilage. **(E)** Safranin O staining of healthy and OA human cartilage (*n* = 10 for each group). **(F,G)** The integrated optical density (IOD) value **(F)** and positive staining area (%) **(G)** of proteoglycan content in healthy and OA human cartilage tissues determined by safranin O staining (*n* = 10 for each group). **(H)** RIP1 immunostaining of healthy and OA human cartilage (*n* = 10 for each group). **(I)** The IOD value of RIP1 immunostaining in healthy and OA human cartilage tissues (*n* = 10 for each group). **(J)** The mRNA level of RIP1 in primary chondrocytes derived from healthy and OA human cartilage tissues measured by qRT-PCR (*n* = 10 for each group). **(K)** Safranin O staining and RIP1 immunostaining of cartilage from sham- and ACLT-operated rats (*n* = 10 for each group). **(L)** Safranin O staining and RIP1 immunostaining of cartilage from 3- and 12-month old rats (*n* = 10 for each group). **(M)** RIP1 immunofluorescent staining of rat chondrocytes treated with or without 10 ng/mL IL-1β for 24 h (*n* = 3 for each group; three independent experiments). **P < 0.01 and ***P < 0.001.

First, to explore whether chondrocyte necrosis existed in human OA cartilage, EBD uptake of the chondrocytes within freshly obtained cartilage tissues of OA patients and healthy donors was determined *in situ* by confocal microscopy. The results showed that obvious EBD-positive staining was observed within the cytoplasm of OA chondrocytes relative to the healthy control, indicating the existence of necrosis (Figures 1A,B). The LDH concentration of the cultural supernatant from OA cartilage explants was also significantly higher than that from healthy cartilage tissues, further demonstrating the injury of chondrocytes and release of intracellular contents from OA cartilage (Figure 1C). TEM was conducted to directly visualize the necrotic morphology of chondrocytes. Chondrocytes exhibiting typical morphological features of necrosis including cell swelling, loss of membrane integrity and interior structures disintegration were presented in OA cartilage, while the cell morphology within healthy cartilage was intact and normal (Figure 1D).

Next, to investigate the role of RIP1 in cartilage degeneration, we examined the expression level of RIP1 in cartilage samples from 10 OA patients and 10 healthy donors. While proteoglycan content was significantly lower in OA cartilage compared to that in healthy control, the expression level of RIP1 was greatly increased as evidenced by immunohistochemical staining (Figures 1E–I). To avoid the interference of inflammatory cell infiltration, the upregulation of RIP1 in OA was further validated in primary chondrocytes derived from healthy and OA cartilage tissues by qRT-PCR (Figure 1J). Moreover, we have demonstrated the same expression trend of RIP1 in normal and OA rat cartilage caused by trauma, as well as spontaneous OA rat cartilage caused by old age (Figures 1K,L). IL-1β stimulation is used as a conventional way to recapitulate the pathological condition of *in vitro* OA cell model. As shown in our results, immunofluorescent staining of RIP1 was greatly enhanced in chondrocytes with IL-1β treatment compared to those without treatment (Figure 1M). The above observations confirm the upregulation of RIP1 during OA progression.

### RIP1 Induces Significant Chondrocyte Death Including Necroptosis and Apoptosis

**FIGURE 2:**
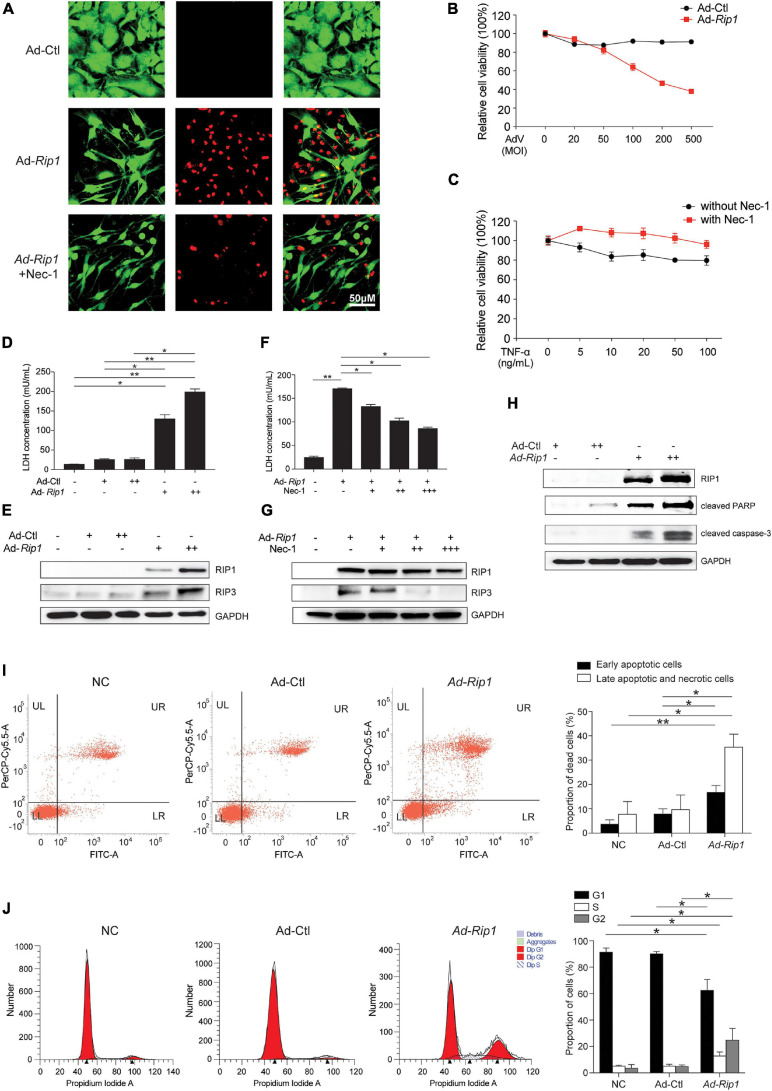
Overexpression of RIP1 in chondrocytes causes necroptosis and apoptosis. **(A)** Live-dead staining of rat chondrocytes treated with Ad-Ctl (100 MOI), Ad-*Rip1*, or Ad-*Rip1* with 50 μM Nec-1 (*n* = 3 for each group). Green fluorescence: live cells; red fluorescence: dead cells. **(B)** Relative cell viability of rat chondrocytes assessed by CCK-8 assay after treatment with indicated MOI of Ad-Ctl or Ad-*Rip1* for 24 h (*n* = 6 for each group). **(C)** Relative cell viability of rat chondrocytes assessed by CCK-8 assay after treatment with indicated doses of TNF-α with or without 50 μM Nec-1 for 24 h (*n* = 6 for each group). **(D)** LDH concentrations in the cultural supernatant of rat chondrocytes treated with Ad-Ctl (100, 200 MOI) or Ad-*Rip1* for 24 h (*n* = 3 for each group; three independent experiments). **(E)** The protein levels of RIP1 and RIP3 detected by western blotting assay in rat chondrocytes treated with Ad-Ctl (100, 200 MOI) or Ad-*Rip1* for 24 h. **(F)** LDH concentrations in the cultural supernatant of rat chondrocytes treated with Ad-Ctl (100 MOI), Ad-*Rip1* or Ad-*Rip1* and Nec-1 (50, 100, and 200 μM) for 24 h (*n* = 3 for each group; three independent experiments). **(G)** The protein levels of RIP1 and RIP3 detected by western blotting assay in rat chondrocytes treated with Ad-Ctl (100 MOI), Ad-*Rip1* or Ad-*Rip1* and Nec-1 (50, 100, and 200 μM) for 24 h. **(H)** The protein levels of RIP1 and cleaved PARP and cleaved caspase-3 detected by western blotting assay in rat chondrocytes treated with Ad-Ctl (100, 200 MOI) or Ad-*Rip1* for 48 h. **(I)** Necrotic and apoptotic rat chondrocytes stained by annexin V and PI and analyzed by flow cytometry after treatment with Ad-Ctl (100 MOI) or Ad-*Rip1* for 24 h, rat chondrocytes without treatment were used as negative control (*n* = 3 for each group; three independent experiments). **(J)** Cell cycle distribution of rat chondrocytes treated with Ad-Ctl (100 MOI) or Ad-*Rip1* for 24 h, rat chondrocytes without treatment were used as negative control (*n* = 3 for each group; three independent experiments). *P < 0.05 and **P < 0.01.

Next, we sought to determine whether the upregulation of RIP1 was sufficient to trigger chondrocyte necroptosis. RIP1-expressing adenovirus vector was constructed and transfected to chondrocytes, the live-dead staining results showed that RIP1 overexpression by adenovirus led to robust chondrocyte death, while application of its small molecule inhibitor Nec-1 significantly reversed that effect (Figure 2A). CCK-8 assay further demonstrated that RIP1 induced impaired chondrocyte viability in a dose-dependent manner (Figure 2B), and Nec-1 effectively blocked TNF-α-mediated decline of chondrocyte viability (Figure 2C). Compared to control adenovirus, loss of cell membrane integrity increased dose-dependently in chondrocytes transduced with RIP1-expresssing adenovirus as indicated by the LDH concentration in the cultural supernatant (Figure 2D). RIP1 overexpression also caused the upregulation of RIP3, the key marker of necroptosis, further supporting the occurrence of necroptosis in chondrocytes induced by RIP1 (Figure 2E). Meanwhile, Nec-1 suppressed RIP1-mediated LDH release and RIP3 upregulation also in a dose-dependent way (Figures 2F,G). These results suggest that RIP1 triggers chondrocyte necroptosis, which can be reversed by its inhibitor Nec-1. Moreover, RIP1 also induced the expression of cleaved PARP and cleaved caspase-3, indicating that RIP1 is involved in the activation of not only necroptosis, but also apoptosis in chondrocytes (Figure 2H). To further clarify RIP1-mediated necroptosis and apoptosis in chondrocytes, flow cytometry assay with annexin V and PI staining was conducted. Both early apoptotic cells (Annexin V-FITC^+^/PI^–^) and late apoptotic/necrotic cells (Annexin V-FITC^+^/PI^+^) were profoundly increased by RIP1 (Figure 2I). Furthermore, flow cytometry was performed to determine the impact of RIP1 on the cell cycle distribution of chondrocytes, and it showed that RIP1 caused significant cell cycle arrest in G2 phase (Figure 2J). Taken together, these findings suggest that upregulation of RIP1 is sufficient to trigger both necroptosis and apoptosis in chondrocytes.

### Enhanced RIP1 Deteriorates ECM Metabolic Homeostasis in Chondrocytes

**FIGURE 3:**
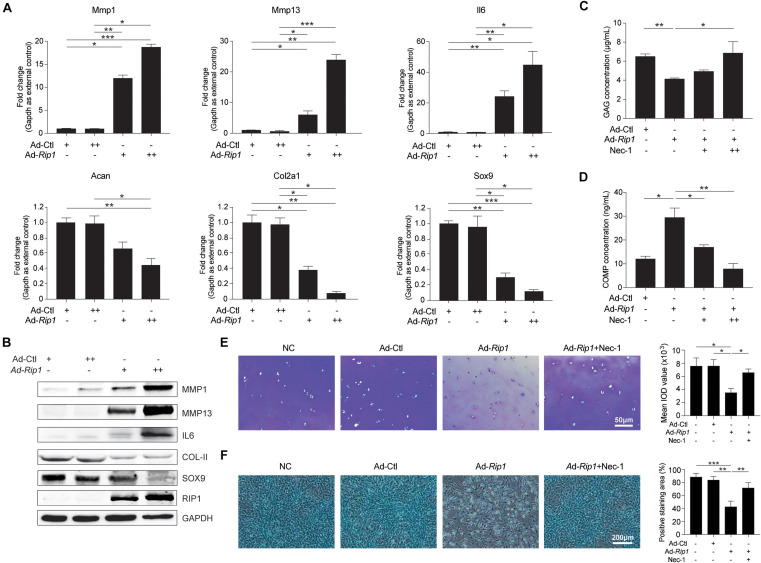
RIP1 promotes catabolism and inhibits anabolism in chondrocytes. **(A)** The mRNA levels of ECM-related biomarkers *Mmp1*, *Mmp13*, *Il6*, *Acan*, *Col2a1*, and *Sox9* in rat chondrocytes treated with Ad-Ctl (100, 200 MOI) or Ad-*Rip1* for 24 h (*n* = 5 for each group; three independent experiments). **(B)** The protein levels of MMP1, MMP13, IL6, COL2A1, SOX9, and RIP1 detected by western blotting assay in rat chondrocytes treated with Ad-Ctl (100, 200 MOI) or Ad-*Rip1* for 24 h. **(C,D)** The concentration of GAG in cartilage explants **(C)** and the concentration of COMP released from the explants **(D)** treated with Ad-Ctl (100 MOI), Ad-*Rip1* or Ad-*Rip1* and Nec-1 (50 and 100 μM) for 10 days (*n* = 6 for each group). **(E)** Toluidine blue staining of the cartilage explants treated with Ad-Ctl (100 MOI), Ad-*Rip1* or Ad-*Rip1* and Nec-1 (100 μM) for 10 days (*n* = 6 for each group). **(F)** Alcian blue staining of the chondrocytes treated with Ad-Ctl (100 MOI), Ad-*Rip1* or Ad-*Rip1* and Nec-1 (100 μM) for 24 h (*n* = 6 for each group). *P < 0.05, **P < 0.01, and ***P < 0.001.

We proceeded to investigate whether upregulation of RIP1 in chondrocytes elicits ECM-related gene expression pattern changes. Ad-*Rip1*-infected chondrocytes displayed increased mRNA levels of catabolic enzymes matrix metalloproteinase 1 (MMP1), MMP13, and proinflammatory cytokines IL6, as well as decreased levels of ACAN, COL2A1, and SOX9 (Figure 3A). The protein levels of MMP1, MMP13, and IL6 were also augmented after RIP1 overexpression, while COL2A1 and SOX9 were significantly downregulated by it (Figure 3B). Next, to evaluate whether RIP1 causes ECM loss *ex vivo*, cartilage disk was harvested from intact human knee cartilage and cultured in the medium containing control adenovirus or RIP1-expressing adenovirus with or without Nec-1 for 10 days. The concentration of matrix content GAG in the cartilage was decreased by RIP1, and this reduction was dose-dependently blocked by Nec-1 treatment (Figure 3C). COMP has been shown to reflect the severity of joint damage in OA and is classified as a biomarker of cartilage degeneration (Lohmander et al., 1994; Clark et al., 1999). The amount of COMP released from the cartilage explant was greatly increased by RIP1, and Nec-1 also inhibited that effect (Figure 3D). Moreover, toluidine blue staining of the cartilage as well as Alcian blue staining of the chondrocytes further demonstrated that RIP1 upregulation caused robust loss of ECM, which could be rescued by Nec-1 (Figures 3E,F). These results demonstrate that RIP1 sabotages the metabolic homeostasis of cartilage by altering the expression of ECM-related genes.

### Intra-Articular Overexpression of RIP1 Induces Chondrocytes Necroptosis and OA-Related Syndromes

**FIGURE 4:**
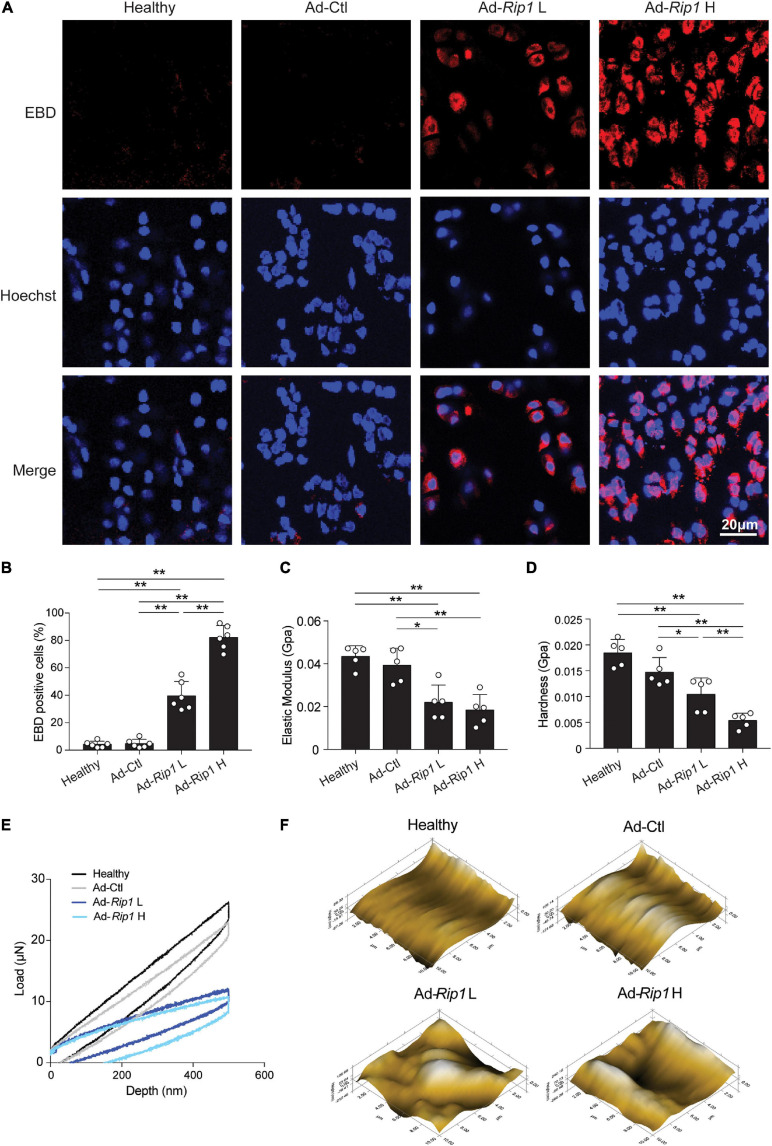
Intra-articular injection of Ad-*Rip1* causes chondrocyte necroptosis and impaired biomechanical properties of rat cartilage. **(A,B)** Representative photomicrographs **(A)** and quantitative data **(B)** of chondral EBD uptake (red) in cartilage from rats intra-articularly injected with Ad-Ctl (5 × 10^8^ pfu) and two different doses of Ad-*Rip1* (10^8^ pfu, 5 × 10^8^ pfu) (*n* = 6 for each group). Cell nuclei were labeled by Hoechst. **(C–F)** Elastic modulus **(C)**, hardness **(D)**, load-displacement curves **(E)**, and microscopic geomorphology **(F)** of cartilage surface from rats intra-articularly injected with Ad-Ctl (5 × 10^8^ pfu) and two different doses of Ad-*Rip1* (10^8^ pfu, 5 × 10^8^ pfu) evaluated by nanoindentation test (*n* = 5 for each group). *P < 0.05 and **P < 0.01.

Next, the contribution of RIP1 to chondrocyte necroptosis and OA pathogenesis was assessed by intra-articular overexpression of RIP1 in rats. One month after injection of Ad-Ctl (5 × 10^8^ pfu) and two different doses of Ad-*Rip1* (10^8^ pfu, 5 × 10^8^ pfu) respectively, frozen sections of rat knee joints from each group were examined by confocal microscopy, and normal rat knee joints were used as negative control. Notably, overexpression of RIP1 by adenovirus vector led to significant loss of chondrocyte membrane integrity in rats, as indicated by increased EBD penetration, which was stronger as the dose increased (Figures 4A,B). Then we investigated the biomechanical properties of the cartilage surfaces from each group. Compared to negative control and Ad-Ctl groups, Ad-*Rip1* injection groups exhibited lower elastic modulus and hardness, and the load-displacement curves further revealed the impaired biomechanical strength of cartilage caused by RIP1 (Figures 4C–E). Moreover, the microscopic geomorphology of the indentation zones in Ad-*Rip1* injection groups appeared to be much rougher than the control groups (Figure 4F).

**FIGURE 5:**
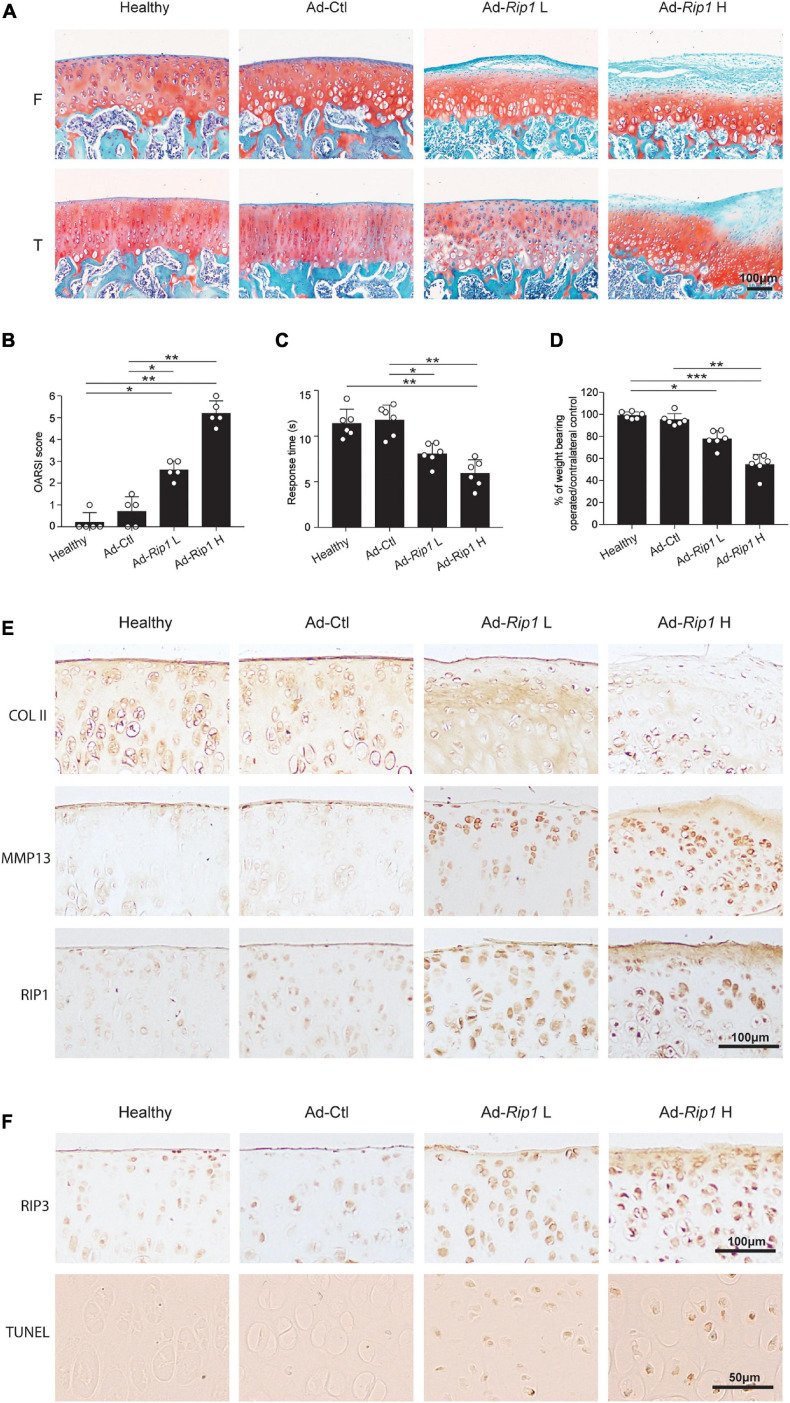
Intra-articular overexpression of RIP1 induces OA-related symptoms in rats. **(A,B)** Safranin O and fast green staining **(A)** and the corresponding OARSI scores **(B)** of the cartilage from rats intra-articularly injected with Ad-Ctl (5 × 10^8^ pfu) and two different doses of Ad-*Rip1* (10^8^ pfu, 5 × 10^8^ pfu) (*n* = 5 for each group) F: femur; T: tibia. **(C)** The pain response times for the rats of each group when placed on the 55°C hotplate meter (*n* = 6 for each group). **(D)** The ratios of weight placed on the hindlimb with adenovirus injection vs. that on the contralateral sham hindlimb for the rats of each group (*n* = 6 for each group). **(E)** COL II, MMP13, and RIP1 immunostaining of the cartilage from rats intra-articularly injected with Ad-Ctl (5 × 10^8^ pfu) and two different doses of Ad-*Rip1* (10^8^ pfu, 5 × 10^8^ pfu) (*n* = 5 for each group). **(F)** RIP3 immunostaining and TUNEL staining of the cartilage from rats intra-articularly injected with Ad-Ctl (5 × 10^8^ pfu) and two different doses of Ad-*Rip1* (108 pfu, 5 × 108 pfu) (*n* = 5 for each group). *P < 0.05, **P < 0.01, and ***P < 0.001.

Next, we sought to determine whether RIP1 overexpression in rat knee joints was sufficient enough to induce OA manifestations. Cartilage destruction condition was examined using safranin O-fast green staining, and then scored using the OARSI grading system. The results revealed that RIP1 overexpression caused significant disruption of articular cartilage relative to control groups (Figures 5A,B). Hotplate analysis and weight bearing test were conducted to evaluate the level of pain for the injected limb of the rats from each group. RIP1 overexpression led to faster response of the limb on hotplate, as well as the imbalance of weight bearing between the injected limb and the contralateral one, and higher dose of RIP1 caused a more significant change, indicating OA-induced pain behavior mediated by RIP1 (Figures 5C,D). The immunohistochemical results showed that RIP1 caused significant downregulation of type II collagen and enhanced expression of MMP13, and gene delivery efficiency of the adenovirus by intra-articular injection was confirmed by increased expression of RIP1 in cartilage (Figure 5E). The status of chondrocyte necroptosis and apoptosis within rat joints was determined by IHC of RIP3 and TUNEL staining, respectively. And the results showed that RIP1 increased both the expression of RIP3 and TUNEL-positive chondrocytes in rat cartilage tissue (Figure 5F). These data suggest that, similar to its function *in vitro*, RIP1 plays an important role in OA by mediating chondrocyte necroptosis and apoptosis, as well as OA-related pathological changes in rat knee joints.

### Inhibition of RIP1 Activity by Nec-1 Significantly Attenuates OA

**FIGURE 6:**
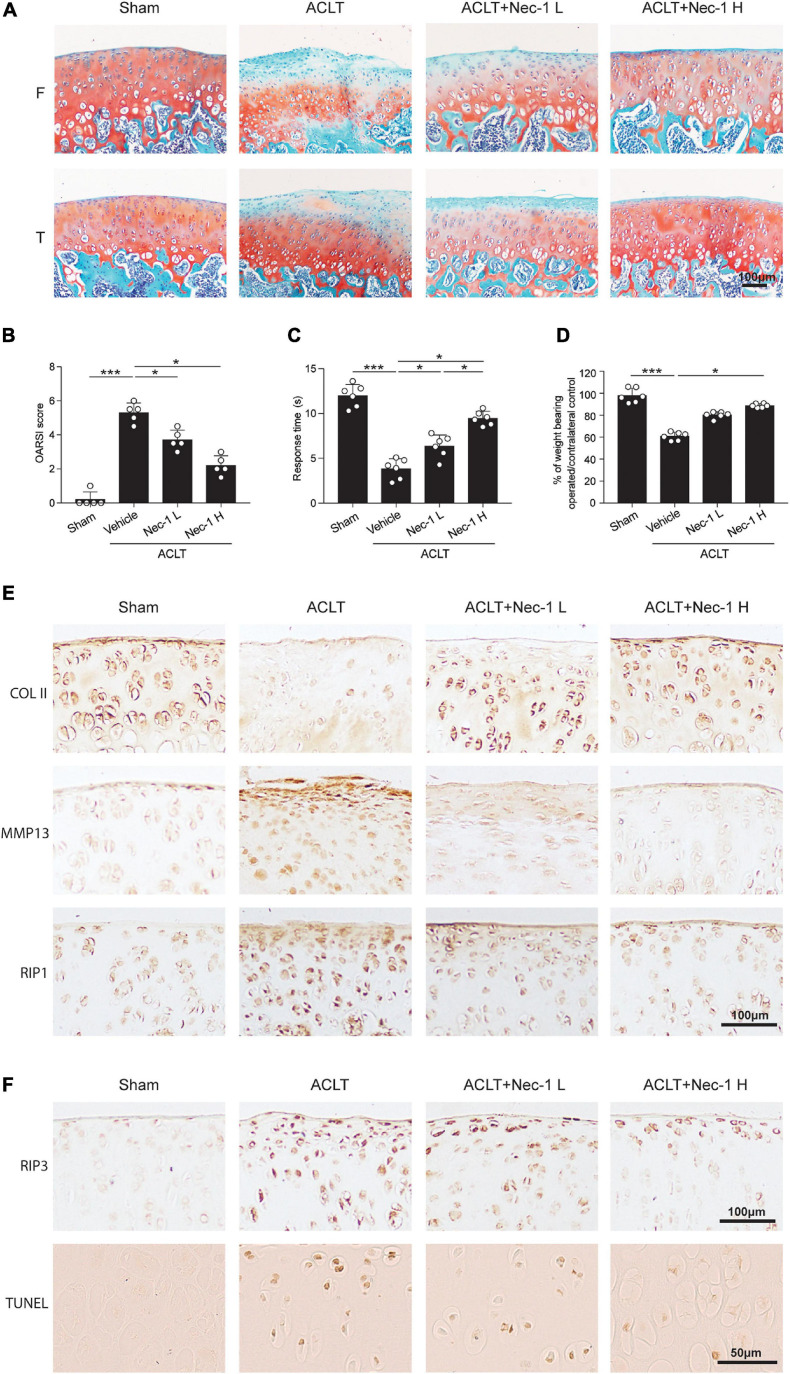
Inhibition of RIP1 enzymatic activity by Nec-1 blocks OA cartilage destruction and the related pain. **(A,B)** Safranin O and fast green staining **(A)** and the corresponding OARSI scores **(B)** of the cartilage from rats with sham or ACLT surgery following by intra-articular injection of vehicle control or two different doses of Nec-1 (0.025 and 0.05 mg/kg) (*n* = 5 for each group) F, femur; T, tibia. **(C)** The pain response times for the rats of each group when placed on the 55°C hotplate meter (*n* = 6 for each group). **(D)** The ratios of weight placed on the operated hindlimb vs. that on the contralateral sham hindlimb for the rats of each group (*n* = 6 for each group). **(E)** COL II, MMP13, and RIP1 immunostaining of the cartilage from rats with sham or ACLT surgery following by intra-articular injection of vehicle control or two different doses of Nec-1 (0.025 and 0.05 mg/kg) (*n* = 5 for each group). **(F)** RIP3 immunostaining and TUNEL staining of the cartilage from rats with sham or ACLT surgery following by intra-articular injection of vehicle control or two different doses of Nec-1 (0.025 and 0.05 mg/kg) (*n* = 5 for each group). *P < 0.05, and ***P < 0.001.

To investigate the effect of inhibiting RIP1 kinase activity by its small-molecule inhibitor Nec-1 on OA progression, a destabilized OA animal model was generated by transecting the ACL in rats. Three days after surgery, we conducted intra-articular injection of vehicle control and two different doses of Nec-1 (0.025 and 0.05 mg/kg) into the operated knee joints of rats respectively. Notably, cartilage ECM loss induced by ACLT was attenuated by Nec-1 injection, and higher concentration of Nec-1 led to a more obvious improvement, as indicated by OARSI scores (Figures 6A,B). Local administration of Nec-1 in ACLT rats also significantly alleviated OA-related pain as assessed by hotplate assay and weight bearing test (Figures 6C,D). Moreover, the expression of type II collagen was significantly higher in Nec-1-treated ACLT rats than the vehicle-treated ones, while the expression of MMP13 was markedly reduced by Nec-1, both of which were in a dose-dependent fashion, indicating protection from articular cartilage degeneration by inhibiting RIP1 activity (Figure 6E). Meanwhile, augmented expression of RIP1 caused by trauma was also blocked by Nec-1 application (Figure 6E). IHC of RIP3 and TUNEL staining results showed that ACLT procedure elicited chondrocyte necroptosis and apoptosis within rat joints, which was reversed by Nec-1 (Figure 6F). Taken together, these results indicate that inhibition of RIP1 activity by Nec-1 in rat articular cavity efficiently protects the chondrocytes from necroptosis and apoptosis, and meliorates OA-related symptoms including cartilage degeneration as well as limb pain during disease progression.

### Analysis of Chondrocyte Transcriptome After RIP1 Overexpression

Since MLKL has been identified as a direct target of necrosome formed by RIP1 and RIP3, and serves as a key component of the necroptosis machinery (Sun et al., 2012; Zhao et al., 2012), we investigated its possible involvement in RIP1-mediated downstream effects using chondrocytes isolated from wild-type and *Mlkl* KO mice. The results showed that ablation of MLKL did not completely block RIP1-induced chondrocyte necroptosis and ECM-related gene expression alterations (Supplementary Figures 1A,B), indicating that MLKL is dispensable for RIP1-induced pathological changes in chondrocytes, and that RIP1 upregulation may potentiate OA progression *via* non-canonical MLKL-independent functions.

**FIGURE 7:**
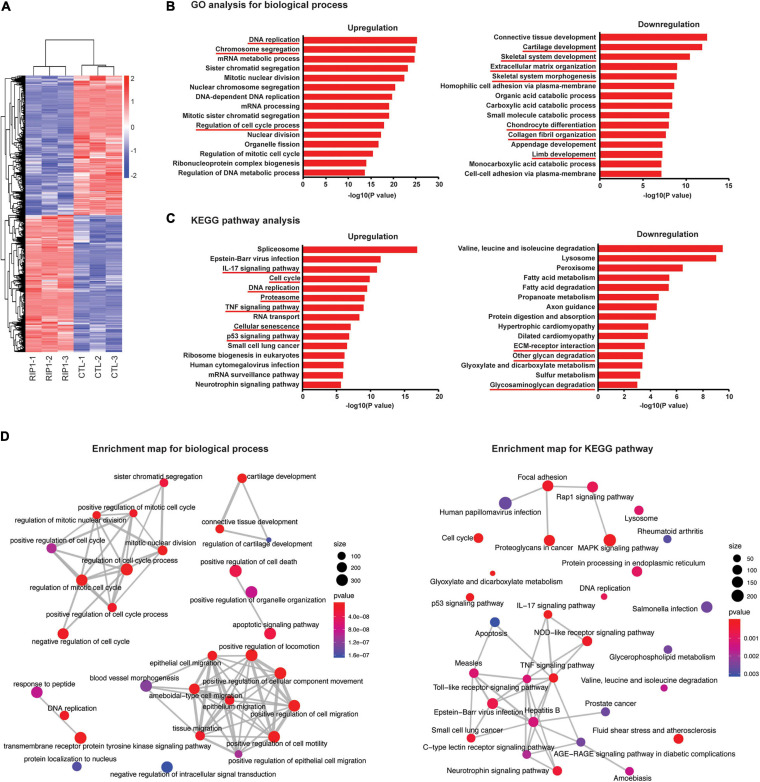
Chondrocyte transcriptome after RIP1 overexpression determined by RNA-seq. **(A)** Heatmap of differential expressed genes in rat chondrocytes treated with Ad-Ctl (100 MOI) or Ad-*Rip1* for 24 h (*n* = 3 for each group). **(B,C)** GO analysis **(B)** and pathway analysis **(C)** for differentially expressed genes of rat chondrocyte transcriptome. **(D)** Enrichment map generated for graphical representations of top 30 enriched biological processes and KEGG pathways.

To identify new molecular targets of RIP1 in chondrocytes, RNA sequencing (RNA-seq) was performed in chondrocytes treated with adenovirus expressing RIP1 or vector control. We found that 9,857 genes were differentially expressed in chondrocytes after RIP1 overexpression (Figure 7A and Supplementary Figure 2). GO analysis indicated that DNA replication, chromosome segregation and regulation of cell cycle process were upregulated, while terms including cartilage development, skeletal system development, ECM organization, skeletal system morphogenesis, chondrocyte differentiation, collagen fibril organization and limb development were downregulated (Figure 7B). Pathway analysis revealed that IL-17 signaling pathway, cell cycle, DNA replication, proteasome, TNF signaling pathway, cellular senescence and p53 signaling pathway were significantly upregulated by RIP1, meanwhile, ECM-receptor interaction, other glycan degradation and glycosaminoglycan degradation were downregulated (Figure 7C). Regulation of cell cycle process and TNF signaling pathway were significantly enriched according to enrichment maps generated for biological processes and KEGG pathways, respectively (Figure 7D). These results underscore the importance of RIP1 in OA by perturbing a series of essential events during disease progression such like cell cycle regulation, chondrocyte differentiation, inflammation and ECM remodeling.

### Identification of BMP7 as the Target of RIP1 in Chondrocytes

**FIGURE 8:**
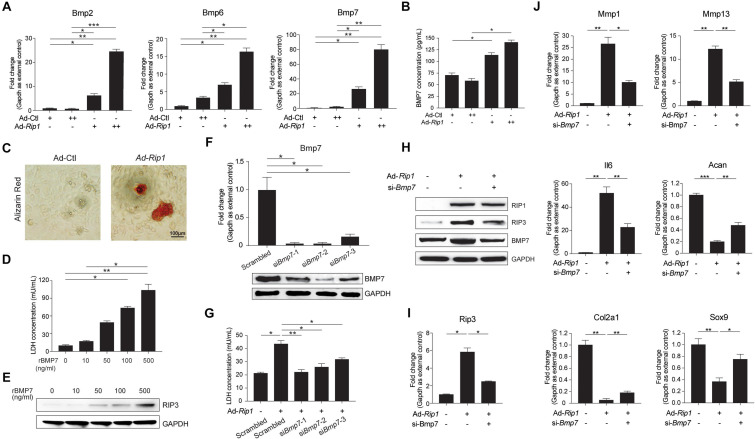
BMP7 mediates RIP1-induced OA pathological signatures in chondrocytes. **(A)** The mRNA levels of *Bmp2*, *Bmp6*, and *Bmp7* in rat chondrocytes treated with Ad-Ctl (100, 200 MOI) or Ad-*Rip1* for 24 h (*n* = 4 for each group; three independent experiments). **(B)** The concentration of secreted BMP7 in the cultural supernatant of rat chondrocytes treated with Ad-Ctl (100, 200 MOI) or Ad-*Rip1* for 48 h (*n* = 3 for each group; three independent experiments). **(C)** Alizarin red staining of rat chondrocytes treated with Ad-Ctl (100 MOI) or Ad-*Rip1* for 48 h (*n* = 4 for each group). **(D)** LDH concentration in the cultural supernatant of rat chondrocytes treated with indicated doses of recombinant BMP7 for 24 h (*n* = 4 for each group). **(E)** The protein level of RIP3 in rat chondrocytes treated with indicated doses of recombinant BMP7 for 24 h. **(F)** The mRNA and protein levels of BMP7 in rat chondrocytes transfected with scrambled or BMP7-targeted siRNAs (*n* = 4 for each group). **(G)** LDH concentration in the cultural supernatant of rat chondrocytes treated with Ad-Ctl (100 MOI) or Ad-*Rip1* following by transfection of BMP7-targeted siRNAs (*n* = 4 for each group). **(H)** The protein levels of RIP1, RIP3, and BMP7 in rat chondrocytes treated with Ad-Ctl (100 MOI) or Ad-*Rip1* following by transfection of BMP7-targeted siRNA. **(I)** The mRNA level of *Rip3* in rat chondrocytes treated with Ad-Ctl (100 MOI) or Ad-*Rip1* following by transfection of BMP7-targeted siRNA (*n* = 4 for each group; three independent experiments). **(J)** The mRNA levels of *Mmp1*, *Mmp13*, *Il6*, *Acan*, *Col2a1*, and *Sox9* in rat chondrocytes treated with Ad-Ctl (100 MOI) or Ad-*Rip1* following by transfection of BMP7-targeted siRNA (*n* = 4 for each group; three independent experiments). *P < 0.05, **P < 0.01, and ***P < 0.001.

Bone morphogenetic protein family is known to play a predominant role in chondrocyte differentiation and ECM remodeling, as well as endochondral ossification, the dysregulation of which are essential processes during OA development (van der Kraan et al., 2010). Previous studies have suggested that multiple BMPs play important roles in chondrocyte biology, including BMP2, BMP6, BMP7, BMP9, and BMP14 (Chen et al., 2004; van der Kraan et al., 2010; Salazar et al., 2016; Thielen et al., 2019), among which BMP2, BMP6, and BMP7 were significantly upregulated in RIP1-overexpressing chondrocytes according to our RNA-seq results (Supplementary Figure 3). By verifying the mRNA levels of BMP2, BMP6, and BMP7 in chondrocytes with or without RIP1 overexpression, we confirmed that the induction level of BMP7 by RIP1 was much higher than the other two (Figure 8A). Therefore, BMP7 appears as a promising candidate that functions downstream of RIP1 in chondrocytes during OA progression. To further demonstrate that RIP1 promoted the expression of BMP7, the concentration of BMP7 in the cultural supernatant of chondrocytes was determined before and after RIP1 overexpression, the result showed that RIP1 increased secreted BMP7 level in a dose-dependent manner (Figure 8B). As BMPs play pivotal roles in the regulation of bone induction, maintenance and repair, we also performed Alizarin red staining to reveal the formation of calcium deposits in chondrocytes, which are indicative of ossification (Lowery and Rosen, 2018; Gooding et al., 2019; Wei et al., 2020). As expected, RIP1-overexpressing chondrocytes showed positive reaction with Alizarin red while control cells were negative, indicating that RIP1 indeed induces endochondral ossification, a crucial process during OA progression (Figure 8C).

To investigate the contribution of BMP7 on chondrocyte necroptosis, we treated the chondrocytes with recombinant BMP7, and it showed that BMP7 triggered chondrocyte necroptosis dose-dependently as indicated by increased LDH concentration in the cultural supernatant and induction of RIP3 in chondrocytes (Figures 8D,E). We proceeded to examine if silencing BMP7 could inhibit the effect of RIP1 on chondrocytes. The gene knockdown efficiency of three siRNAs targeting BMP7 was confirmed at both mRNA and protein levels (Figure 8F), and all of them inhibited LDH release from the chondrocytes induced by RIP1 to varying degrees (Figure 8G). We chose siRNA-2 with the best knockdown efficiency for subsequent detection, and silencing of BMP7 with this siRNA in chondrocytes significantly inhibited the upregulation of RIP3 mediated by RIP1, further supporting the participation of BMP7 in RIP1-mediated chondrocyte necroptosis (Figures 8H,I). Whether silencing BMP7 blocks the regulatory effect of RIP1 on ECM-related genes expression was also determined, and the results showed that the induction of MMP1, MMP13, and IL6 by RIP1 was greatly suppressed after BMP7 silencing, while the downregulation of ACAN, COL2A1, and SOX9 by RIP1 were significantly restored (Figure 8J). Thus, these data demonstrate that BMP7 mediates the inductive effect of RIP1 on chondrocyte necroptosis and cartilage ECM degeneration, contributing to the onset of OA.

## Discussion

Osteoarthritis is characterized by progressive destruction of articular cartilage, resulting in significant disability. Since articular cartilage depends solely on its resident cells, chondrocytes, to maintain the ECM, compromising of chondrocyte function and survival would lead to the failure of the articular cartilage (Pitsillides and Beier, 2011; Aicher and Rolauffs, 2014). Chondrocyte death is the most common pathological feature in OA, and literatures revealed that there is a definite correlation between chondrocyte apoptosis and cartilage damage (Thomas et al., 2007; Ryu et al., 2012; Hosseinzadeh et al., 2016). However, contradictory reports exist on the relative contribution of chondrocyte apoptosis in the pathogenesis of OA, and reducing apoptosis by blocking the apoptotic pathways might increase necrosis (Holler et al., 2000; Han et al., 2011). Necroptosis, as a new form of programmed cell death, is critically involved in vital physiological and pathological processes including embryonic development, host responses to bacterial and viral infection, tissue injury and inflammation, and has been intensively investigated in various disease conditions (Pasparakis and Vandenabeele, 2015; Galluzzi et al., 2017; Weinlich et al., 2017). Although the possible involvement of chondrocyte necroptosis in OA has been suggested, direct evidences are still lacking. Recent studies immunohistochemically analyzed the expression of necroptosis markers RIP3, MLKL, and p-MLKL to prove the existence of necroptosis in degenerated human and murine cartilage (Riegger and Brenner, 2019; Stolberg-Stolberg et al., 2020). However, it remains unclear whether chondrocyte necroptosis is the inducer of cartilage destruction or its byproduct, and the expression status of another important necroptosis marker, RIP1, in OA clinical samples has not been assessed. In this study, typical necrotic cell morphology in OA clinical samples was observed *in situ* for the first time. And we investigated the expression status of RIP1 in OA and its contribution to the disease using not only experimental OA rat models, but also human OA cartilage, which is closer to the clinical disease status. Our findings indicate that upregulation of RIP1 is essentially involved in OA pathogenesis.

Receptor-interacting protein kinase 1 has emerged as a crucial regulator in various human diseases such like cancers, neurodegeneration, autoimmune, and inflammatory diseases (Ofengeim and Yuan, 2013; Liu et al., 2015; Ofengeim et al., 2017; Wang et al., 2018; Tao et al., 2020). While the kinase activity of RIP1 mediates the activation of RIP3 and caspase-8 to promote necroptosis and apoptosis respectively, RIP1 also serves as a signaling scaffold to prevent the activation of RIP3 and caspase-8 in a kinase-independent manner, implying the tissue-specific complicated role of RIP1 (Degterev et al., 2019). Our study suggests that RIP1 upregulation in chondrocytes causes both necroptosis and apoptosis, as well as changes in ECM metabolism-related gene expression patterns. More importantly, we have provided the first *in vivo* evidence that initiation of necroptosis by intra-articular overexpression of RIP1 alone is sufficient to trigger typical osteoarthritic manifestations including impaired mechanical properties of cartilage, pain, ECM loss and subsequent cartilage destruction in rats. Therefore, activation of RIP1 represents a key factor to promote OA pathogenesis. Given the fact that current treatments for OA act only on symptoms and cannot alleviate or cure OA, and that RIP1 functions upstream of RIP3 and MLKL, RIP1 could be a valid target to modulate cartilage degeneration.

Numerous studies have indicated that Nec-1, the specific small molecule inhibitor of RIP1, effectively delayed disease progression in an extensive list of animal models such as acute ischemic brain, heart, kidney, and eye injuries, but the effects of Nec-1 on OA progression have not been evaluated comprehensively (Smith et al., 2007; Trichonas et al., 2010; Northington et al., 2011; Chavez-Valdez et al., 2012; Linkermann et al., 2012). A previous study has shown that Nec-1 abolished the increases of MMP3, MMP13, and ADAMTS5 expression induced by IL-1β in mouse chondrocytes, and suppressed cartilage catabolism in a destabilized medial meniscus (DMM) mouse model (Liang et al., 2018). However, the dose-dependent chondro-protective effect of Nec-1 has not been evaluated *in vivo*, and its therapeutic efficiency on limb pain-related behaviors, the major symptom of OA, were not assessed either. Herein, our study showed that Nec-1 abolished RIP1-mediated necroptosis, apoptosis and ECM disruption in chondrocytes and cartilage explants without chondrocyte cytotoxicity, and local injection of Nec-1 efficiently alleviated trauma-induced OA pathogenic signatures in a dose-dependent manner. We also provided the first evidence that Nec-1 potently ameliorated OA-related pain in rats as demonstrated by hotplate analysis and weight bearing test. Nevertheless, further studies are required to investigate other Nec-1 targets, and the treatment efficacy of Nec-1 in larger preclinical animal models of OA, as well as its extra-articular and systemic side effects before entering clinical trials.

Initially, necroptosis seemed to be following the typical pathway, and most studies have focused on manipulating the RIP3-MLKL cascade to regulate necroptosis and diverse disease processes. Deficiency of MLKL prevents necroptosis in multiple cell types, including tumor cells, macrophages, and fibroblasts (Sun et al., 2012; Zhao et al., 2012; Wu et al., 2013; Wang et al., 2014). But with recent studies reporting diverse pathways and outcomes, the necroptosis signaling has become a lot more interesting and intricate. There is a need to further understand the alternative tissue-specific interactions of necroptosis signaling molecules. In this study, we demonstrate that RIP1 plays a MLKL-independent role in cartilage by evoking chondrocytes necroptosis and disrupting ECM metabolism homeostasis in the absence of MLKL, and that BMP7 is also essential for linking RIP1 to chondrocyte death and the resultant structural and functional defects of cartilage. These findings highlight a distinct regulation form of RIP1-dependent necroptosis in chondrocytes compared with that in many other cell types, in which it requires MLKL. Nevertheless, the participation of other BMP family members during RIP1-induced OA progression cannot be excluded, as BMP family is shown to act in collaboration during bone morphogenesis. Our results showed that although to a lesser degree, BMP2 and BMP6 were also upregulated by RIP1 in chondrocytes. Therefore, the possible involvement of other BMPs in RIP1-mediated OA manifestations needs to be explored in the future study.

As a kinase, RIP1 induces necroptosis and apoptosis following its enzymatic activation by directly phosphorylating its downstream targets RIP3 and caspase-8, respectively (Degterev et al., 2019). Therefore, the potential mechanism regarding how RIP1 positively regulates the expression of BMP7 to trigger chondrocyte necroptosis and apoptosis might be an indirect modulation *via* an intermediate effector. RIP1 is known to activate NF-κB, which has been demonstrated to regulate the transcription of BMP family members like BMP2 and BMP4 (Feng et al., 2003; Fukui et al., 2006; Zhu et al., 2007). There were also putative NF-κB response elements within the promoter region of BMP7, but whether these NF-κB response elements are functional in chondrocytes or NF-κB promotes BMP7 gene transcription through these response elements remains to be further explored. And more evidences are needed to support the hypothesis that RIP1 upregulates BMP7 *via* activating NF-κB in chondrocytes.

BMP7, also known as osteogenic protein-1, is a member of the transforming growth factor-β (TGF-β) superfamily that acts, *via* its downstream Smad1/5/8, as endogenous counter-regulator of TGF-β1 signaling (Meng et al., 2013). BMP7 controls the development and maintenance of multiple physiological processes in the human body, and its aberrant expression has found to be associated with a variety of pathologic conditions (i.e., incomplete fracture healing, the development of bone metastases in cancers, renal fibrosis, obesity, and OA) (Boon et al., 2011). Endochondral ossification is an essential process not only for physiological skeletal growth but also for the development of OA. Previous studies have revealed that BMP7 induced hypertrophy and endochondral ossification in rat knees by mediating chondrocyte differentiation (Garciadiego-Cázares et al., 2015), and that upregulation of BMP7 in plasma and synovial fluid is related to progressive joint damage determined by radiographic and symptomatic changes, indicating that BMP7 might serve as a biochemical parameter for determining disease severity in primary knee OA (Honsawek et al., 2009; Schmal et al., 2015). However, evidences also showed that controlled release of low concentrated BMP7 leads to the decrease of inflammation and matrix degradation markers as well as the induction of matrix synthesis in osteoarthritic chondrocytes (Gavenis et al., 2011), suggesting a pleiotropic role of BMP7 in the regulation of chondrocyte fate. Therefore, careful titration of BMP7 inhibition might be a possible avenue for the prevention of OA or the treatment of this disease at early stages.

In conclusion, we demonstrate that upregulation of RIP1 contributions to OA pathogenesis by mediating chondrocyte necroptosis and ECM destruction *via* BMP7, a newly identified downstream target of RIP1, in addition to MLKL. These findings reveal a previously unappreciated mode of necroptosis and pave the way for future research investigating RIP1-BMP7 blockade as a novel therapeutic approach for OA.

## Data Availability Statement

The datasets presented in this study can be found in online repositories. The names of the repository/repositories and accession number(s) can be found below: GEO; GSE165219.

## Ethics Statement

The studies involving human participants were reviewed and approved by the Ethics Committee of Peking University Third Hospital. The patients/participants provided their written informed consent to participate in this study. The animal study was reviewed and approved by the Institutional Animal Care and Use Committee of Peking University Health Science Center. Written informed consent was obtained from the individual(s) for the publication of any potentially identifiable images or data included in this article.

## Author Contributions

JC, YA, and XZ contributed to the conception and design of the study. JC, XD, XF, and PY performed the experiments. JC, CC, QL, and JZ collected the data. JC and YJ contributed to data analysis and interpretation. JC and XH drafted the work and revised it critically for intellectual content. YA and XZ reviewed the manuscript and supervised the project. All authors contributed to the article and approved the submitted version.

## Conflict of Interest

The authors declare that the research was conducted in the absence of any commercial or financial relationships that could be construed as a potential conflict of interest.
