# Supplementary material for: RIP1 Perturbation Induces Chondrocyte Necroptosis and Promotes Osteoarthritis Pathogenesis via Targeting BMP7
Source: Front Cell Dev Biol. 2021 Apr 16;9:638382. doi: 10.3389/fcell.2021.638382 (PMC8085605; doi:10.3389/fcell.2021.638382)
Supplement: Supplementary file 1 [file Data_Sheet_1.DOCX]

Supplementary Material

# Supplementary Figures

**
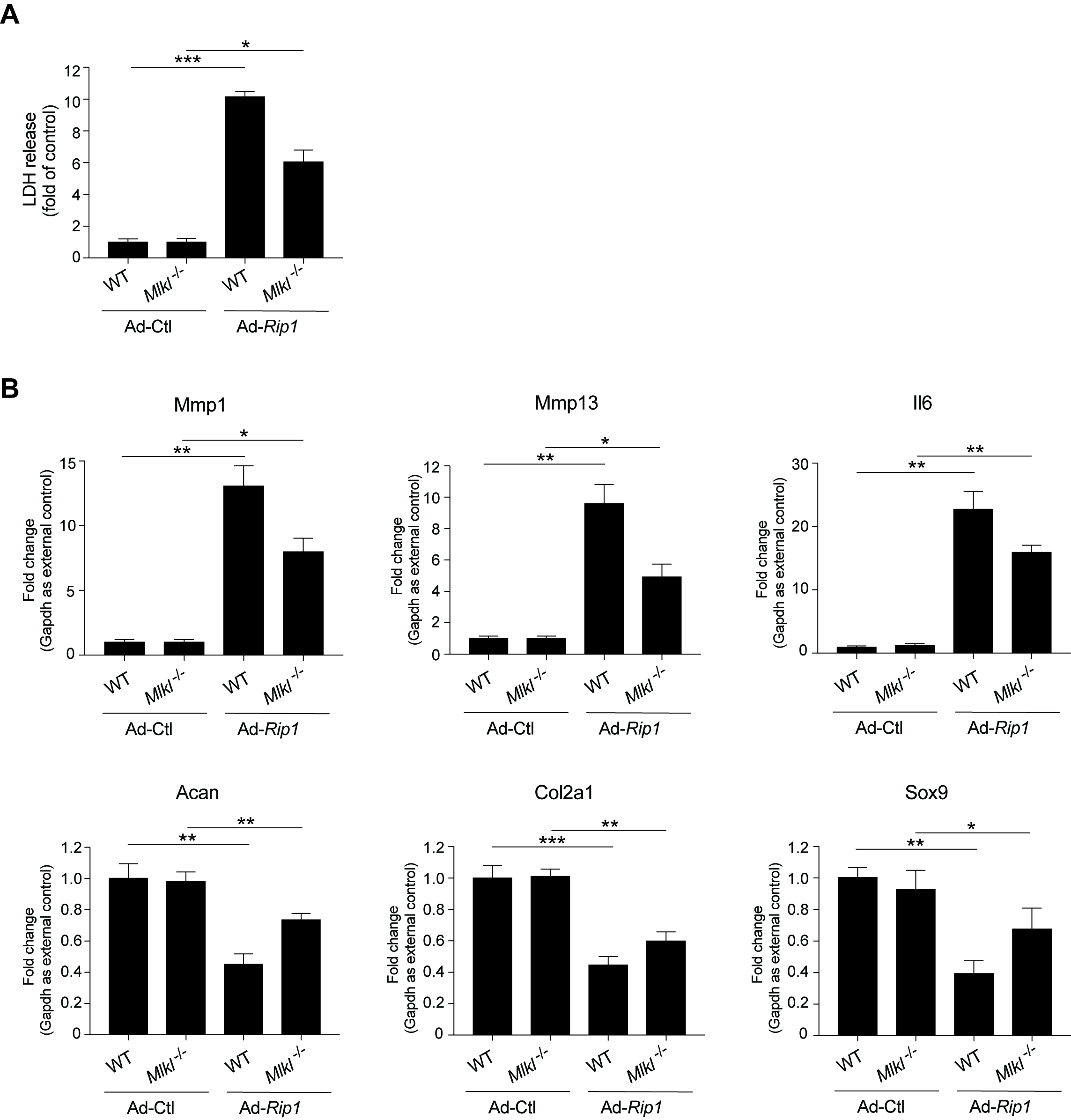
**

**Supplementary Figure 1. RIP1 induces chondrocyte necroptosis and abrogates ECM metabolism balance** **in the absence of MLKL.** **(A)** LDH concentrations in the cultural supernatant of wild-type and *Mlkl* KO mouse chondrocytes treated with Ad-Ctl (100 MOI) or Ad-*Rip1* for 24 hrs (n=4 for each group). **(B)** The mRNA levels of *Mmp1*, *Mmp13*, *Il6*, *Acan*, *Col2a1* and *Sox9* in wild-type and *Mlkl* KO mouse chondrocytes treated with Ad-Ctl (100 MOI) or Ad-*Rip1* for 24 hrs (n=4 for each group; three independent experiments). ^*^*P* < 0.05, ^**^*P* < 0.01, ^***^*P* < 0.001.


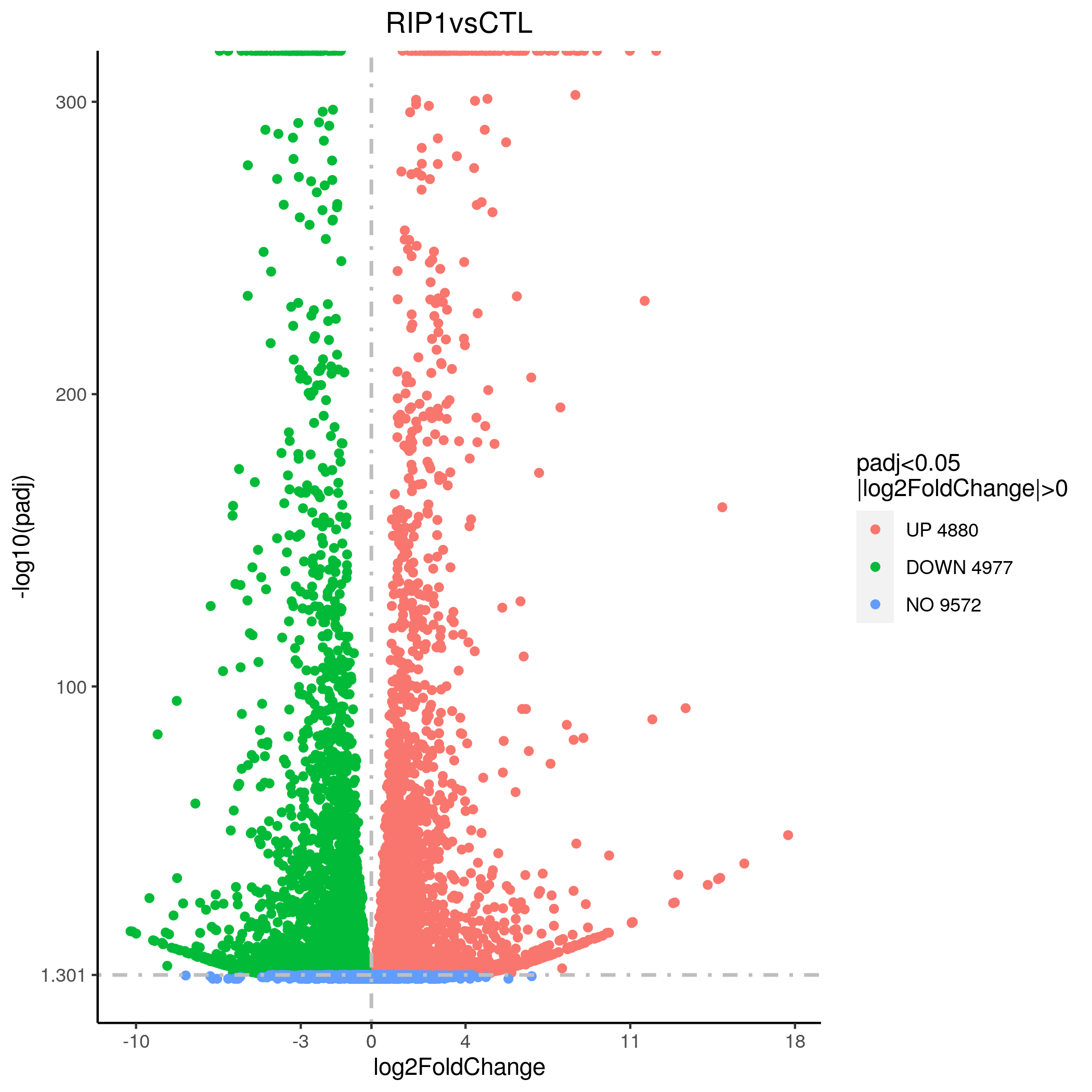


**Supplementary Figure 2. A total of 9857 genes were differentially expressed in chondrocytes after RIP1 overexpression.** Volcano plot of the differential expressed genes in rat chondrocytes treated with Ad-Ctl (100 MOI) or Ad-*Rip1* for 24 hrs (n=3 for each group).

**
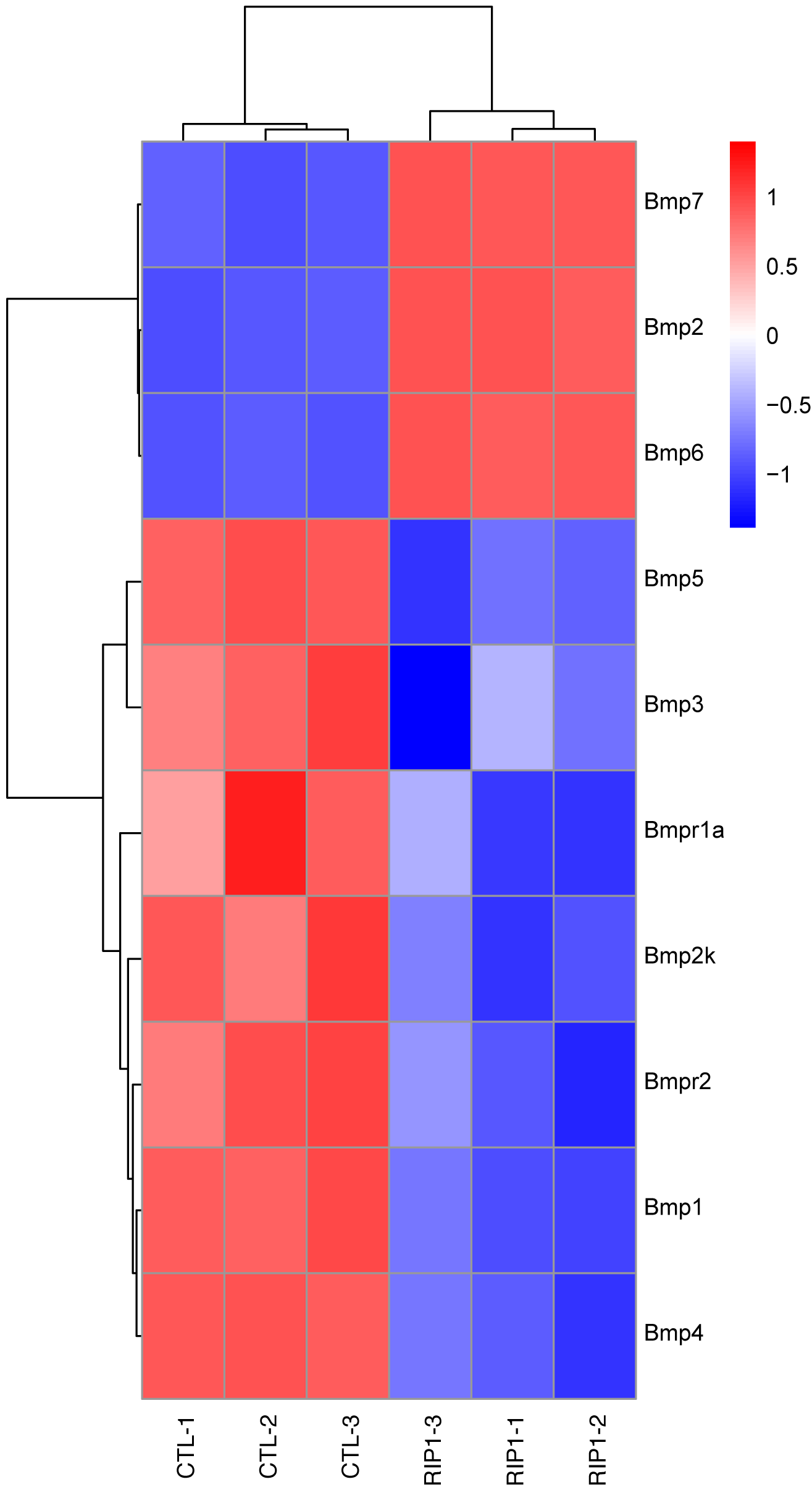
**

**Supplementary Figure 3. Ten BMP family members were differentially expressed in chondrocytes after RIP1 overexpression.** Heatmap of differentially expressed BMP family members in rat chondrocytes treated with Ad-Ctl (100 MOI) or Ad-*Rip1* for 24 hrs.

# Supplementary Tables

**Supplementary Table 1. Characteristics of specimens from patients with OA**

| No. | Age/gender | ICRS grade | Joint | Weight (kg) | Height (m) | BMI (kg/m^2^) |
| --- | --- | --- | --- | --- | --- | --- |
| 1 | 58/F | 4 | Knee | 65 | 1.56 | 26.71 |
| 2 | 68/F | 4 | Knee | 74 | 1.52 | 32.03 |
| 3 | 55/F | 4 | Knee | 81 | 1.50 | 36.00 |
| 4 | 55/F | 4 | Knee | 79 | 1.58 | 31.65 |
| 5 | 64/F | 4 | Knee | 71 | 1.62 | 27.05 |
| 6 | 66/F | 4 | Knee | 79 | 1.64 | 29.37 |
| 7 | 66/F | 4 | Knee | 62 | 1.55 | 25.81 |
| 8 | 61/M | 4 | Knee | 76 | 1.73 | 25.39 |
| 9 | 73/M | 4 | Knee | 66 | 1.56 | 27.12 |
| 10 | 67/M | 4 | Knee | 79 | 1.70 | 27.34 |

ICRS, International Cartilage Repair Society; BMI, Body Mass Index

**Supplementary Table 2. The qRT-PCR primer sequences for ECM-related genes**

| **Primer sequences for the detection of target genes expression in rats** | | | | | | |
| --- | --- | --- | --- | --- | --- | --- |
| **Gene name** | **Forward (5’-3’)** | | **Reverse (5’-3’)** | | |  |
| **Mmp1** | GCAGTGTGACACACATTGAC | | GATAACCTGGATCCATGGAC | | |  |
| **Mmp13** | CTGCGGTTCACTTTGAGGAC | | ACAGCATCTACTTTGTCGCC | | |  |
| **Il6** | AAGCCAGAGTCATTCAGAGC | | TTAGCCACTCCTTCTGTGAC | | |  |
| **Acan** | CCACTGGAGAGGACTGCGTAG | | GGTCTGTGCAAGTGATTCGAG | | |  |
| **Col2a1** | CACCGCTAACGTCCAGATGAC | | GGAAGGCGTGAGGTCTTCTGT | | |  |
| **Sox9** | AGGAAGCTGGCAGACCAGTA | | ACGAAGGGTCTCTTCTCGCT | | |  |
| **Bmp2** | ACCCTTTGTATGTGGACTTCAGTGATG | | CTATGGCATGGTTGGTGGAGTTCAG | | |  |
| **Bmp6** | CAGCAGCAACAATCGCAACAGAC | | GAGTTGTAGAGATCCAGCATGAAGAGC | | |  |
| **Bmp7** | CTGTATGTTAGCTTCCGAGACCTTGG | | GTGGCGTTCATGTAGGAGTTCAGAG | | |  |
| **Rip3** | ATCATCTGACACCTTGGCTG | | AGACTGTAGGCCTGTCATTG | | |  |
| **Gapdh** | GCAAGTTCAACGGCACAG | | GCCAGTAGACTCCACGACA | | |  |
|  | | | | | | |
|  | |  | |  | | |
| **Primer sequences for the detection of target genes expression in human** | | | | | | |
| **Gene name** | | **Forward (5’-3’)** | | **Reverse (5’-3’)** |  |  |
| **MMP1** | | TGAACTCGGCCATTCTCTTG | | AACGTCCATATATGGCTTGG |  |  |
| **MMP13** | | ACTGAGAGGCTCCGAGAAATG | | GAACCCCGCATCTTGGCTT |  |  |
| **IL6** | | TTCGGTACATCCTCGACGGCATC | | CAGCTCTGGCTTGTTCCTCACTAC |  |  |
| **ACAN** | | ACTCTGGGTTTTCGTGACTCT | | ACACTCAGCGAGTTGTCATGG |  |  |
| **COL2A1** | | CCAGATGACCTTCCTACGCC | | TTCAGGGCAGTGTACGTGAAC |  |  |
| **SOX9** | | AGCGAACGCACATCAAGAC | | CTGTAGGCGATCTGTTGGGG |  |  |
| **GAPDH** | | GCACCGTCAAGGCTGAGAAC | | TGGTGAAGACGCCAGTGGA |  |  |

| **Primer sequences for the detection of target genes expression in mice** | | |  |  |
| --- | --- | --- | --- | --- |
| **Gene name** | | **Forward (5’-3’)** | **Reverse (5’-3’)** | |
| **Mmp1** | | AACTACATTTAGGGGAGAGGTGT | GCAGCGTCAAGTTTAACTGGAA | |
| **Mmp13** | | CTTCTTCTTGTTGAGCTGGACTC | CTGTGGAGGTCACTGTAGACT | |
| **Il6** | | TACCACTTCACAAGTCGGAGGC | CTGCAAGTGCATCATCGTTGTTC | |
| **Acan** | | CAGATGGCACCCTCCGATAC | ACACACCTCGGAAGCAGAAG | |
| **Col2a1** | | TCCCTCGGAAAAACTGGTGG | CCACCGTTCATGGTCTCTCC | |
| **Sox9** | | GTGCAAGCTGGCAAAGTTGA | TGCTCAGTTCACCGATGTCC | |
| **Gapdh** | | GGTGGAGCCAAAAGGGTCAT | TCGTGGTTCACACCCATCAC | |
